# Effects of a mobile health nutrition intervention on dietary intake in children who have autism spectrum disorder

**DOI:** 10.3389/fped.2023.1100436

**Published:** 2023-02-15

**Authors:** Tanja V. E. Kral, Lauren O’Malley, Kelsey Johnson, Teresa Benvenuti, Jesse Chittams, Ryan J. Quinn, J. Graham Thomas, Jennifer A. Pinto-Martin, Susan E. Levy, Emily S. Kuschner

**Affiliations:** ^1^ Department of Biobehavioral Health Sciences, School of Nursing, University of Pennsylvania, Philadelphia, PA, United States; ^2^ Department of Psychiatry, Perelman School of Medicine, University of Pennsylvania, Philadelphia, PA, United States; ^3^ Center for Injury Research and Prevention, Children's Hospital of Philadelphia, Philadelphia, PA, United States; ^4^ CHDI Management/CHDI Foundation, Princeton, NJ, United States; ^5^ School of Medicine, Duke University, Durham, NC, United States; ^6^ Weight Control and Diabetes Research Center, Brown University School of Medicine and The Miriam Hospital, Providence, RI, United States; ^7^ Department of Biostatistics, Epidemiology and Informatics, Perelman School of Medicine, University of Pennsylvania, Philadelphia, PA, United States; ^8^ Department of Pediatrics, Children's Hospital of Philadelphia, Philadelphia, PA, United States; ^9^ Departments of Psychiatry and Radiology, Children's Hospital of Philadelphia, Philadelphia, PA, United States

**Keywords:** mobile health intervention, technology, nutrition, children, autism

## Abstract

**Background:**

Children who have Autism Spectrum Disorder (ASD) show preferences for processed foods, such as salty and sugary snacks (SSS) and sugar-sweetened beverages (SSB), while healthier foods, such as fruits and vegetables (FV), are consumed less. Innovative tools are needed that can efficiently disseminate evidence-based interventions and engage autistic children to improve their diet.

**Aim:**

The aim of this 3-month randomized trial was to test the initial efficacy of a mobile health (mHealth) nutrition intervention on changing consumption of targeted healthy (FV) and less healthy foods/beverages (SSS, SSB) in children who have ASD, ages 6–10, who were picky eaters.

**Methods:**

Thirty-eight parent-child dyads were randomly assigned to either an intervention (technology) group or a wait list control (education) group. The intervention included behavioral skills training, a high level of personalization for dietary goals, and involved parents as “agents of change.” Parents in the education group received general nutrition education and the dietary goals but did not receive skills training. Children's intake was assessed at baseline and at 3 months using 24-hour dietary recalls.

**Results:**

While there were no significant group-by-time interactions (*P *> 0.25) for any of the primary outcomes, we found a significant main effect of time for FV intake (*P *= 0.04) indicating that both groups consumed more FV at 3 months (2.58 *± *0.30 servings/day) than at baseline (2.17 *± *0.28 servings/day; *P *= 0.03). Children in the intervention group who consumed few FV at baseline and showed high engagement with the technology increased their FV intake by 1.5 servings/day (*P *< 0.01). Children's taste/smell sensitivity significantly predicted their FV intake (*P *= 0.0446); for each unit of *lower* taste/smell sensitivity (indicating greater sensory processing abnormalities), FV intake increased by 0.13 *±* 0.1 servings/day.

**Discussion:**

This mHealth intervention did not yield significant between-group differences for changing consumption of targeted foods/beverages. Only children who consumed few FV at baseline and highly engaged with the technology increased their FV intake at 3 months. Future research should test additional strategies to expand the intervention's impact on a wider range of foods while also reaching a broader group of children who have ASD. This trial was registered at clinicaltrials.gov as NCT03424811.

**Clinical Trial Registration:** This study was registered at clinicaltrials.gov as NCT03424811.

## Introduction

Children who have Autism Spectrum Disorder (ASD)^1^ are five times more likely to have mealtime challenges (4–6) and be picky eaters (7–10), which in part has been attributed to restrictive and ritualistic behaviors and heightened sensory sensitivity (7). Picky eating among autistic youth has been shown to not be associated with a lack of appetite as many parents report that their children show a healthy appetite for the foods that they like (7), which often include highly processed foods (9–13). Consumption patterns that favor energy-dense, nutrient-poor foods and beverages in lieu of healthier options put children on the autism spectrum at an increased risk for excess weight gain. In fact, children who have ASD show a four-fold increased risk for overweight and obesity (14) and are over three times more likely to develop metabolic syndrome (15) than children with neurotypical development.

Children on the autism spectrum have been reported to show preferences for highly processed foods, such as salty and sugary snacks (SSS) and sugar-sweetened beverages (SSB), while healthier foods, such as fruits and vegetables (FV), are consumed less often (9–13). There is strong evidence for an independent role of SSB intake (16–18) and increased snacking frequency (19–22) in the promotion of obesity in youth. Increased FV consumption has been shown to reduce dietary energy density (23, 24), moderate energy intake (25, 26), and play an important role in the prevention and treatment of childhood obesity (27, 28). Reducing consumption of SSB and SSS and increasing consumption of FV in children are therefore considered important targets for intervention.

The pronounced core impairments of social and communication skills and presence of restricted and repetitive behaviors in children who have ASD complicate traditional treatment options for healthy eating available to children with neurotypical development. While behavioral interventions are considered effective treatments overall and, in particular, for selective eating in children who have ASD (29–31), it is often behavioral therapists, and not parents or caregivers, who work with children one-on-one in highly structured clinical settings, which are highly resource dependent and less generalizable to everyday life without structured mealtimes. Parents are often removed from the treatment initially and then trained to re-enter mealtimes as discharge approaches [see Sharp and colleagues (31) for a review of the most common intensive multidisciplinary interventions]. Evidence suggests that treatments that involve family members as treatment providers are effective and may be more applicable to the home environment (32, 33). A small number of studies exist aimed at teaching caregivers, during in-person sessions, behavior modification strategies which they can use to address feeding problems in their children. While some of these interventions indicated a high degree of social validity and caregiver satisfaction, many showed limited success in changing child mealtime behaviors or dietary variety, included small sample sizes, and suffered from high attrition rates (34–38). Barriers to parent-directed interventions include high time commitment and transportation costs, shortage of trained professionals, and lack of childcare (39). New and innovative tools are needed that are scalable, can efficiently disseminate evidence-based parent interventions, and effectively engage children who have ASD.

The use of mobile technology is rapidly increasing in children, including for those on the autism spectrum, across all ages. Children who have ASD, in particular, are engaging with mobile devices on a daily basis and technology has been shown to help with their learning and communication by providing structure and dependability and opportunities for visual learning (40, 41). Research has shown that multisensory interactions and the ability to individualize instructions are some of the features that can assist children who have ASD when working with technology (42) and smart technologies are effective tools for improving social, functional, and communications skills (40, 43). We aimed to harness the lure of technology by developing an innovative, evidence-based mobile health (mHealth) nutrition intervention to teach children who have ASD about healthy eating and to motivate them to make healthy food choices in their daily lives. mHealth technologies provide opportunities for remote coaching and skills training, which has proven to be an effective and efficient training tool for parents of autistic youth (44, 45).

Core behavior change strategies that have been used in family-based nutrition and childhood obesity prevention research in children with neurotypical development include the specification of target behaviors, self-monitoring, goal setting, stimulus control, positive parenting strategies, and promotion of self-efficacy and self-management skills (46, 47). Parents and caregivers act as important “agents of change” for promoting healthy eating among their children. There are parallels in the application of similar core behavior strategies in providing supports for autistic children (48, 49) and many parents are already being trained in these behavior change strategies to address behavioral and communication challenges their children experience (50, 51). Our study is among the first to empirically test the efficacy of incorporating these same core behavior change strategies into a mHealth nutrition intervention to affect changes in the intake of targeted healthy and less healthy foods and beverages in children on the autism spectrum who are picky eaters.

The aim of this 3-month randomized controlled trial was to test the initial efficacy of a mHealth nutrition intervention on changing consumption of targeted healthy (FV) and less healthy foods (SSS) and beverages (SSB) in children on the autism spectrum who were picky eaters. We hypothesized that, by the end of the intervention, children in the technology (intervention) group, but not children in the education (wait list control) group, would show a significant increase (expressed as % change from baseline) in intake of FV and a decrease in calories consumed from SSS and SSB.

## Methods

### Study design

In this exploratory 3-month trial, parent-child dyads were randomly assigned to either a mHealth intervention group (technology group) or a wait list control group (education group) using a randomized block design to produce groups that were comparable in weight status. Parent trainings in both groups were matched for in-person contact time. All study visits were conducted in families' homes to reduce burden and increase child comfort. The study protocol, including screening and recruitment procedures, was approved by the Institutional Review Boards of the University of Pennsylvania and The Children's Hospital of Philadelphia (CHOP). Parents and children were asked to provide voluntary informed consent (parents) and assent (children) to participate in the study by signing the consent and assent forms. During the informed consent/assent process, families were informed that they will be randomized into one of two groups (technology or education group) for the duration of the study. Families were also informed that if they were assigned to the education group, they would be given access to the mobile app for free after their participation in the study ended.

### Study population

Participants for this study included boys and girls with ASD, ages 6–10 years, and their parent or legal guardian (referred to as “parent” for ease of use). This age range is consistent with ages that children would engage in similar technology. Parents had to be the children's primary caregiver (i.e., person responsible for grocery shopping and/or feeding). For this exploratory trial, we recruited a well-defined, homogenous group of children to determine the efficacy of this intervention. Only children without significant intellectual disability (see definition in *Recruitment and Screening Process*) were enrolled to increase the likelihood of comprehension and engagement with the technology. Children with a range in weight were included to explore if the intervention is equally effective in children with normal weight, overweight, and obesity.

A power analysis was conducted (PASS software, Version 11, NCSS LLC, Kaysville, UT) for the primary aim using 3-month changes in intake of targeted healthy foods (FV) as primary outcome variable. The estimated mean (± SD) for baseline FV intake (2.57 ± 1.20 servings/day) was derived from our pilot work with children on the autism spectrum (52). Based on this estimate, a sample size of 46 children with an attrition rate of 10% yielded 80% power to detect a statistically significant increase in FV intake of 1.1 servings/day (or 43%) in the intervention group relative to the control group at an alpha level of 0.05 based on a 2-sample t-test. The magnitude of this increase is comparable to that achieved in behavioral interventions in children with neurotypical development (28, 53) and represents ∼one-fifth of the Recommended Dietary Allowance (RDA) of FV for children of that age (54).

### Recruitment and screening process

Recruitment of study participants was carried out in collaboration with the CHOP Center for Autism Research (CAR). Our multi-pronged recruitment plan utilized the following strategies: (a) CAR's research study page of its website, social media accounts, and email listserv; (b) the CHOP Recruitment Enhancement Core, which leverages recruitment *via* the electronic medical record system; and (c) community-based organizations.

Interested families completed a telephone screening. Parents provided information about their child's age, sex, height, weight, autism diagnosis, medical and developmental history, and medication use and provided verbal authorization per the Health Insurance Portability and Accountability Act (HIPAA) for the phone screening and to have their child's medical information and previous diagnostic evaluations reviewed. Parents were asked to complete the Picky Eating subscale of the Child Feeding Questionnaire (CFQ) (55), which consisted of the following items: (1) “My child's diet consists of only a few foods”; (2) “My child is unwilling to eat many of these foods that our family eats at mealtimes”; (3) “My child is fussy / picky about what she eats.” The items on this subscale have shown adequate internal consistency (*α* = 0.85). Parents were asked follow-up questions to confirm that a child's pickiness was related to the intervention's targeted healthy and less healthy foods to ensure that the intervention goals would be relevant. Picky eating was confirmed if parents endorsed at least two out of the three items on this subscale.

To be included in the study, children had to be between ages 6 to 10 years; fluent in English; have an ASD diagnosis using the DSM-IV-TR or DSM-5 criteria (56, 57) and cognitive skills within the low average (or higher) range with IQ scores of ≥80 and comparable verbal ability; meet the definition of picky eater with pickiness related to the intervention's targeted healthy and less healthy foods; and have access to a mobile device. Medical records were reviewed to confirm documentation of ASD diagnosis by an expert clinician (i.e., developmental pediatrician, clinical psychologist) as well as cognitive and language ability at a level sufficient for comprehension of and engagement with the mHealth technology. When specific IQ or language assessment scores were not available in medical records, school records such as Individualized Education Program documents were reviewed by the expert clinician, and descriptions of skills across academic domains and educational goals were used as a proxy.

Children were excluded from participation in the study if they had moderate-severe hearing/visual or motor impairment (e.g., were non-ambulatory); were taking antipsychotic medications which may be associated with uncontrolled eating; were on a special diet (e.g., gluten/casein-free diet); or had underweight [i.e., body mass index (BMI)-for-age <5th percentile].

### Technology intervention group

#### Description of the behavioral intervention

Parents and children in the technology group received the mHealth nutrition intervention plus training in behavior change strategies. The intervention incorporated core behavior change strategies that have been tested extensively in family-based behavior modification research, including obesity prevention trials (46), and were familiar to families with children who have ASD (58, 59). The unique features of this mHealth intervention were that it (1) reinforced healthy food choices in autistic youth by using behavioral strategies tailored to the specific needs and learning styles of children on the autism spectrum (e.g., visual depictions, concrete descriptions with “scripts”, and routines for abstract concepts), (2) included a high level of personalization to align dietary goals with individual food preferences and sensory sensitivities, and (3) involved parents as “agents of change.” Specifically, children were reinforced for making healthy food choices while limiting less healthy food choices in their daily lives by earning points towards a prize. Targeted *healthy* foods included fresh, canned, and frozen FV. Parents were instructed to omit energy-dense toppings and sauces on FV. Targeted *less healthy* foods included SSS (e.g., all types of chips, popcorn, pretzels, party mixes, ice cream, candy, cookies, cakes/pies, sweet rolls, pastries) and SSB (e.g., sugar-sweetened sodas, fruit drinks/punches and fruit juices, sport drinks, and energy drinks).

The intervention also included behavioral training for children *via* an interactive nutrition education game to facilitate healthier food choices. This involved a “Nutrition Ninja” virtual character, which was directed by the parent and interacted by the child to set and reinforce dietary goals. The inclusion of this virtual character who acted positively towards the child aimed to make the child feel comfortable with technology-mediated communication and offer support for performing the desired behavior, consistent with the Proteus effect (60–62). The goals were very prescriptive and were tailored to children's food preferences and sensory sensitivities by letting them customize dietary targets (e.g., add dip to a vegetable, eat a vegetable raw or cooked, have it served hot or cold). Even small goals, such as touching or smelling a novel food before tasting it, were encouraged. Children received frequent visual and personalized feedback and positive reinforcement to support their unique learning styles. The individual training components for parents and children are summarized in Table 1.

**Table 1:** Training components for parents and children in the intervention group.

**Training type**	**Training components**
**Parent training**	
Behavioral skills training	• Specifying target behaviors and goal setting • Self-monitoring • Stimulus control • Positive reinforcement • Self-efficacy
Nutrition training	• Explanation of nutritional goals and targeted foods and beverages^a^ • Training on how to present feeding opportunities during meal and snack times • Substitute unhealthy target foods and beverages for healthier options • Strategies to overcome resistance in children trying new foods using differential attention and positive reinforcement • Strategies for promoting children's intake of healthy target foods and limiting intake of unhealthy target foods and beverages daily for 3 months
Technology training	• Explanation of the layout and functions of the mHealth technology • Setting dietary goals, selecting rewards, and awarding points • Adding custom foods • Reviewing child's progress, goals, and rewards
**Child training**	
Behavioral skills training	• Specifying target behaviors and goal setting • Positive reinforcement
Nutrition training	• Explanation of the health benefits of fruits and vegetables and undesirable properties of sugary drinks and salty and sugary snacks • Explanation of nutritional goals and targeted foods and beverages • Explanation of “Go” and “Whoa” foods and beverages
Technology training	• Explanation of the layout and functions of the mHealth technology • Setting dietary goals and selecting a reward • Learning how to play the educational Nutrition Ninja game • Communicating with the Nutrition Ninja • Reviewing their progress

^a^ This was the only training component which participants in the waitlist control (education) group received.

#### Description of the mHealth technology

The technology-based intervention, a mobile application, was developed in collaboration with Skyless Game Studios, a Philadelphia-based software company. We used an iterative design process for prototype development, testing and refining of the intervention during which stakeholders (i.e., parents and caregivers of children with autism and children with autism) and technology developers were engaged and asked to provide continuous feedback on the functionality and acceptability of the intervention during the development process.

**Figure 1:**
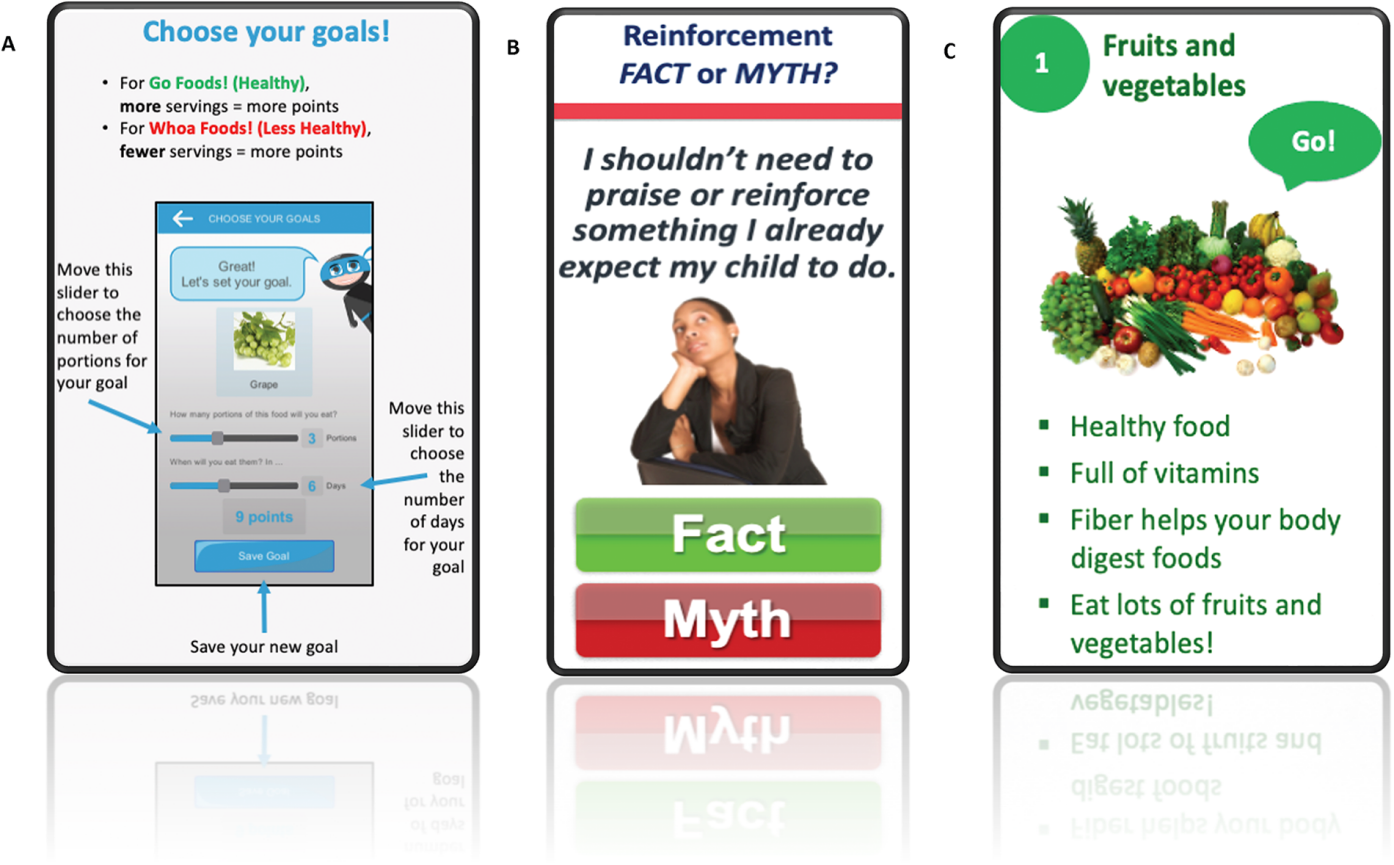
Sample screenshots of the mobile health (mHealth) intervention including goal setting (panel **A**), parent behavioral skills training (panel **B**), and child nutrition training (panel **C**).

During the baseline study visit, trained research staff assisted families with downloading and installing the mobile application onto their smartphones or tablets. They also provided families with a brief technology tutorial which explained to the parent how (1) to view, create, and edit rewards and goals; (2) view their child's progress and award points for eating and drinking goal foods/beverages; (3) add or edit custom foods; and (4) view and send messages to the child. The tutorial explained to the child how to (1) choose their goals; (2) select their reward; (3) send messages to the “Ninja” (i.e., parent) about a food they ate or ask for a food or drink for their next meal; (4) view their progress and awarded points; and (5) play the Nutrition Ninja educational game. Parents and children were also instructed to view a training module, which was built into the app and explained the nutritional targets of the intervention and provided behavioral skills training. Throughout the training, parents and children completed quizzes which reinforced the training content. Parents and children were encouraged to engage with the mHealth technology on a daily basis for 3 months to promote children's intake of the healthy target foods and limit their intake of the less healthy target foods and beverages. Examples of targeted healthy and less healthy target foods were incorporated in the mHealth technology. Figure 1 provides examples of screenshots from the mHealth intervention. The mHealth technology provided research staff with an activity log, which they accessed regularly. For families who showed no activity with the mHealth technology for some time, research staff followed up with them *via* email (after 2 weeks) or a phone call (after 3 weeks) to assess if they had any technical difficulties with the mobile app and reminded them of the goals of the study.

### Education control group

Parents in the Education group received a printed handout which provided general education about healthy eating and explained the nutritional goals and targeted foods and beverages but did not provide any skills training. Parents were encouraged to promote children's intake of healthy target foods and limit intake of unhealthy target foods daily for 3 months. Examples of targeted healthy and less healthy target foods were included on the handout. Families were offered access to the mHealth intervention after they completed the study.

Families in both groups were instructed to try increasing their children's intake of FV and decreasing their intake of SSS and SSB for the 3-month study duration. They were not instructed on what to purchase and make available to their children specifically because we wanted to give families the flexibility to tailor the food and beverage choices to their children's preferences and sensory sensitivities.

### Assessment of child dietary intake

Children's intake was assessed at baseline (before the first study visit) and at the end of the 3-month intervention using the telephone-based 24-hour dietary recall method; the gold standard for self-reported intake (63). Each assessment consisted of three unannounced recalls (two weekdays, one weekend day), conducted by research dietitians from the CHOP and Penn Dietary Assessment Unit at the Center for Human Phenomic Science. Dietitians were blinded to families' group assignment. During each recall, they asked parents, with help from their child, to describe all foods and beverages consumed by their children during the prior 24 h and to provide detailed information about portion sizes and preparation method.

Individual food items were manually coded to derive SSS, SSB and water intake. Data were analyzed for the primary outcomes measures which included daily FV intake (servings/day) and calories consumed per day from SSB and SSS using the University of Minnesota Nutrition Coordinating Center's Food and Nutrient Database. All data were averaged across the three days.

### Assessment of child weight status

During both study visits (i.e., at baseline and at the end of the intervention), children had their heights and weights measured using a digital scale (SECA 876, Chino, CA) and portable stadiometer (SECA 217, Chino, CA). Measurements were taken in triplicate by a trained staff member in children's homes with children wearing light clothing (no shoes). Child age- and sex-specific BMI percentiles and z-scores were calculated using the CDC Growth Charts 2000 (64). Children were classified as normal-weight (BMI-for-age 5–84th percentile), overweight (BMI-for-age 85–94th percentile), or obese (BMI-for-age ≥95th percentile) (65).

### Questionnaires

#### Demographic questionnaire

During the baseline home visit, parents completed a demographic questionnaire which included questions about their age, race/ethnicity, marital status, education, household income, and food security status. Families' food security status was assessed using the 6-item short form of the U.S. Household Food Security survey module (66). The survey's raw score, which is the sum of affirmative responses to the six survey questions, describes four levels of food security: high food security (i.e., no indications of food access problems or limitations); marginal food security (i.e., 1–2 indications of food access problems such as anxiety over food sufficiency or shortage of food in the house); low food security (i.e., reports of reduced quality, variety, or desirability of diet); and very low food security (i.e., reports of multiple indications of disrupted eating patterns and reduced food intake) (67). This short form of the U.S. Household Food Security survey module has shown to have acceptable conceptual validity, specificity (99.5%) and sensitivity (85.9%) for determination of overall food insecurity for households with children (66) as well as good internal consistency (Cronbach's alpha = 0.86) (68).

#### Sensory profile

Parents were also asked to complete the 38-item Short Form Sensory Profile (69), which measures children's sensory processing. For this study, we limited the analysis to the taste/smell sensitivity domain only, which is comprised of the following items: “Avoids certain tastes or food smells that are typically part of children's diets,” “Will only eat certain tastes,” “Limits self to particular food textures/temperatures,” and “Picky eater, especially regarding food textures.” Parents were asked to indicate, on a 5-point scale ranging from “always” (1) to “never” (5), the frequency with which their child responds to these sensory experiences. Using a reverse scoring system, lower scores corresponded to more frequent child behavioral responses. Children were categorized into “Typical Performance,” “Probable Difference,” or “Definite Difference” in oral sensory sensitivity based on the classifications specified by Dunn (70). Construct validity has been established for the Short Form of the Sensory Profile (71) and the tool has been shown to have acceptable reliability and excellent validity (72).

## Statistical analysis

Data were analyzed using the statistical software SAS (Version 9.4; SAS Institute Inc, Cary, NC). The Shapiro-Wilk test was used in combination with histograms and summary statistics to confirm normal distribution of all outcome variables. Outcome variables included intake of fruits and vegetables (servings/day; with and without French Fries); vegetables (servings/day; with and without French Fries); fruit (servings/day); sweet and savory snacks (kcal/day); sweet snacks (kcal/day); savory snacks (kcal/day); sugar-sweetened beverages (kcal/day and fl oz/day); and water (fl oz/day). Participants' baseline demographic, clinical and anthropometric characteristics and dietary intake were compared between the technology and education group using Chi-Square and t-tests for categorical and continuous variables, respectively.

To test the primary aim, an intent-to-treat approach was used. We constructed separate linear mixed effects models for each outcome measure with an unstructured covariance matrix to account for within-subject variance. The effects of time (baseline, 3-month follow-up) and treatment group were included in all models. A time-by-group interaction was included to assess changes in outcomes over time by group. Furthermore, we created adjusted models which controlled for children's age, BMI z-score, and level of taste/smell sensitivity.

Secondary analyses assessed the intervention utilizing a dose-response approach. We explored the extent to which level of engagement with the technology impacted participants' dietary outcomes. In this exploratory analysis, we assessed both planned behavior (i.e., completion of nutrition training and setting goals) and performed behavior (i.e., number of points earned for meeting goals). We then categorized children into those with a high planned engagement (i.e., children who completed the nutrition training and set at least one goal) and those with low or no engagement (i.e., children who completed the nutrition training but did not set any goals or children who neither completed the nutrition training nor set any goals). In terms of performed behavior, we categorized children's engagement with the technology using a tertile split (low/no, medium, high). Analyses tested the effect of children's level of engagement with the technology and the time-by-engagement interaction on dietary outcomes. Analyses were conducted with participants in the education (control) group included in the statistical model; their level of engagement was set to “low/no engagement” because they did not have access to the technology.

Additional exploratory analyses examined the effects of the intervention on changes in dietary outcomes with the sample stratified into low or high consumers at baseline based on a median split in intake. Stratified analyses were conducted for analyses of both the primary predictor (intervention group) and secondary predictor (engagement level) with and without controlling for participants' age, BMI z-score, and level of taste/smell sensitivity. All analyses were evaluated at the alpha level of 0.05 and are considered exploratory. Results are presented as model-based means (± standard error).

## Results

### Child and parent characteristics

Child and parent demographic and anthropometric characteristics are shown in Table 2. The majority of children were male (95%), and many were White (68%); 13% were Hispanic. Half of the children had either overweight (16%) or obesity (34%), respectively. Among parents, 84% were married; 42% had a college degree; and 61% had household incomes greater than $100,000. Groups did not differ significantly in any demographic or anthropometric characteristics (*P* > 0.14), nor in their baseline intake of the target foods (*P* > 0.099).

**Table 2:** Child and parent demographic and clinical characteristics by group.

**Characteristic**	**Technology group (*N* = 19)**	**Nutrition education group (*N* = 19)**	***P*-value**
***Children***			
Age (years), *mean ± SD*	8.9 *± *1.2	8.4 *± *1.4	0.26
Sex*, male/female, n (%)*	18 (94.7%)/1 (5.3%)	18 (94.7%)/1 (5.3%)	1.00
**Race, *n (%)***			
Asian	0	2 (10.5%)	0.16
Black or African American	4 (21.1%)	1 (5.3%)	
White	11 (57.9%)	15 (78.9%)	
More than one race	3 (15.8%)	1 (5.3%)	
Unknown	1 (5.3%)	0	
**Ethnicity, *n (%)***			
Hispanic or latino	4 (21.1%)	1 (5.3%)	0.20
Not hispanic or latino	12 (63.1%)	17 (89.5%)	
Unknown	3 (15.8%)	1 (5.3%)	
**Weight status, *n (%)***			
BMI z-score, *mean ± SD*	0.8 ± 1.36	0.6 ± 1.35	0.64
BMI-for-age percentile, *mean ± SD*	68.1 ± 34.2	63.1 ± 37.2	0.67
Has normal weight	10 (52.6%)	9 (47.4%)	0.82
Has overweight	2 (10.5%)	4 (21.1%)	
Has obesity	7 (36.8%)	6 (31.6%)	
**Sensory *p*rofile (score), *mean ± SD***			
Taste/Smell Sensitivity	9.7 *± *4.9	11.9 *± *4.0	0.14
**Taste/smell sensitivity classification, *n (%)***			
Typical *p*erformance	4 (21.1%)	5 (26.3%)	0.48
Probably difference	3 (15.8%)	6 (31.6%)	
Definite difference	12 (63.2%)	8 (42.1%)	
**Parents**			
**Academic degree, *n (%)***			
High school	3 (15.8%)	4 (21.1%)	1.00
College	8 (42.1%)	8 (42.1%)	
Master’s	6 (31.6%)	6 (31.6%)	
Doctorate	2 (10.5%)	1 (5.3%)	
**Household income, *n (%)***			
<$50,000	2 (10.5%)	4 (21.1%)	0.73
$50,000–$100,000	4 (21.1%)	5 (26.3%)	
$100,000–$150,000	5 (26.3%)	5 (26.3%)	
>$150,000	8 (42.1%)	5 (26.3%)	
**Marital status, *n (%)***			
Single	2 (10.5%)	0	0.54
Married, remarried	15 (78.9%)	17 (89.5%)	
Divorced, separated, widowed	2 (10.5%)	2 (10.5%)	
**Food security status, *n (%)***			
High/marginal food security	18 (94.7%)	16 (84.2%)	0.74
Low food security	0	2 (10.5%)	
Very low food security	1 (5.3%)	1 (5.3%)	

### Efficacy of mHealth intervention on changing target dietary behaviors

Table 3 shows participants' mean intake of FV, SSS, and SSB (primary dietary outcomes) at baseline and at 3 months (end of intervention) by group. There were no significant group-by-time interactions (*P* > 0.25) for any of the primary dietary outcomes. There were also no significant main effects of group for intake of FV, SSS, SSB (*P* > 0.15) or time for intake SSS and SSB (*P* > 0.83). We did find a statistically significant main effect of time for FV intake (*P* = 0.04) indicating that all participants, irrespective of random assignment, on average, consumed significantly more FV at the end of the intervention (2.58 *± *0.30 servings/day) than at baseline (2.17 *± *0.28 servings/day; *P* = 0.03). This change over time in FV intake remained significant after adjusting the model for children's age, BMI z-score and taste/smell sensitivity. When analyzing fruit intake and vegetable intake as separate outcomes and including or excluding French fries in the vegetables category, the main effect of time was not statistically significant (*P* > 0.08). We also explored potential changes in water intake over the course of the intervention but did not find a significant group-by-time effect (*P* = 0.99) or main effects of group or time (*P* > 0.21) on participants' water intake.

**Table 3:** Model-based mean (*±* SEM) intake of primary dietary outcomes at baseline and month 3 (end of intervention).

**Intake**	**Technology (*N* = 19)**		**Nutrition education (*N* = 19)**		**Results from mixed-model analysis**	**Between-group comparison at baseline**
	**Baseline (mean *±* SEM)**	**Month 3 (mean *±* SEM)**	**Baseline (mean *±* SEM)**	**Month 3 (mean *±* SEM)**		
**Fruits and vegetables**						
Fruits and vegetables, *servings/day*	1.79 ±* *0.40	2.16 ± 0.43	2.56 ± 0.40	3.01 ±* *0.41	Group*Time: *P* = 0.82; Group: *P* = 0.15; Time: *P* = 0.04	*P* = 0.15
Fruits, *servings/day*	0.82 ±* *0.36	0.84 ± 0.40	1.48 ± 0.36	1.54 ±* *0.37	Group*Time: *P* = 0.93; Group: *P* = 0.18; Time: *P* = 0.84	*P* = 0.18
Vegetables (french fries included), *servings/day*	0.97 ± 0.20	1.26 ± 0.25	1.08 ± 0.20	1.45 ± 0.21	Group*Time: *P* = 0.83; Group: *P* = 0.55; Time: *P* = 0.08	*P* = 0.66
Vegetables (french fries excluded), *servings/day*	0.71 ± 0.21	0.84 ± 0.25	0.95 ± 0.21	1.29 ± 0.21	Group*Time: *P* = 0.53; Group: *P* = 0.19; Time: *P* = 0.19	*P* = 0.32
**Salty and sugary snacks**						
Salty and Sugary snacks, *kcal/day*	401.6 ± 49.0	420.8 ± 58.4	456.9 ± 49.0	385.7 ± 51.0	Group*Time: *P* = 0.25; Group: *P* = 0.87; Time: *P* = 0.50	*P* = 0.42
Sugary snacks, *kcal/day*	252.8 ± 38.2	225.3 ± 45.7	251.9 ± 38.2	244.3 ± 39.9	Group*Time: *P* = 0.74; Group: *P* = 0.85; Time: *P* = 0.56	*P* = 0.99
Savory snacks, *kcal/day*	148.8 ± 34.8	195.3 ± 41.8	205.0 ± 34.8	141.9 ± 36.3	Group*Time: *P* = 0.06; Group: *P* = 0.98; Time: *P* = 0.77	*P* = 0.22
**Beverages**						
Sugar-sweetened beverages, *fl oz/day*	7.8 ± 1.6	7.6 ± 1.7	7.1 ± 1.6	7.3 ± 1.6	Group*Time: *P* = 0.75; Group: *P* = 0.83; Time: *P* = 0.95	*P* = 0.78
Water, *fl oz/day*	16.1 ± 3.1	17.5 ± 3.5	21.5 ± 3.1	22.9 ± 3.2	Group*Time: *P* = 0.99; Group: *P* = 0.21; Time: *P* = 0.45	*P* = 0.24

### Individual differences in response to nutrition intervention

Overall, the results also remained consistent when adding participants' BMI z-score, age, or taste/smell sensitivity as covariates to the statistical models. We, however, did find that taste/smell sensitivity significantly predicted children's intake of FV when French Fries were included (*P* = 0.0446); for each unit of *lower* taste/smell sensitivity (indicating greater sensory processing abnormalities), estimated mean FV intake increased by 0.13 *±* 0.1 servings/day. We also found a significant effect (*P* = 0.03) of BMI z-score on intake of SSB indicating that for each unit increase in children's baseline BMI z-score, estimated mean SSB intake increased by 1.86 ± 0.8 fl oz/day during the study.

Exploratory stratified analyses revealed that among children who were high consumers of savory snacks at baseline, those in the education group showed a significant *decrease* in calories consumed from savory snacks over time (296 ± 37 kcal/day vs. 165 ± 37 kcal/day; *P* = 0.02) while children in the technology group showed a trend for a significant *increase* in calories consumed from savory snacks over the course of the intervention (286 ± 43 kcal/day vs. 439 ± 61 kcal/day; *P* = 0.06; Group*Time: *P* = 0.007).

### Efficacy of technology-based intervention by engagement

In this exploratory analysis, we assessed the extent to which level of participants' engagement with the technology impacted their dietary outcomes by examining both planned behavior (i.e., completion of nutrition training and setting goals) and performed behavior (i.e., number of points earned for meeting goals).

**Figure 2:**
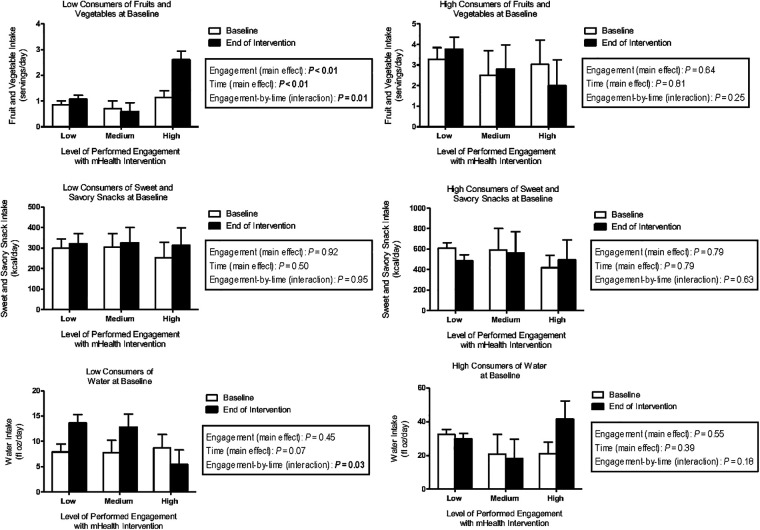
Model-based means (±SEM) of fruit and vegetable intake (servings/day), sweet and savory snack intake (kcal/day), and water intake (fl oz/day) for children in the technology group (*n* = 19) by level of performed engagement (low, medium, high) with the technology and consumption status (low, high) at baseline and at the end of the intervention.

Exploratory stratified analyses revealed that among children who were low consumers of FV (with French Fries excluded) at baseline, those who showed high performed engagement with the technology-based intervention significantly increased their FV intake by 1.5 servings/day over the course of the intervention (engagement-by-time: *P* < 0.01) even when adjusting for children's age, BMI z-score and taste/smell sensitivity (Figure 2).

With respect to savory snacks, findings related to performed and planned engagement were consistent with those seen when using treatment group as predictor. That is, among children who were high consumers of savory snacks at baseline, those who were engaged with the technology exhibited an increase in calories consumed from savory snacks, while those who were not engaged with the technology exhibited a decrease in savory snacks (performed engagement-by-time interaction: *P* = 0.01; planned engagement-by-time interaction: *P* = 0.02).

Further, we found a statistically significant planned (*P* = 0.04) and performed (*P* = 0.03) engagement-by-time interaction for water intake in our stratified analysis. That is, among children who were low consumers of water at baseline, those who showed little planned or performed engagement with the technology exhibited a significant *increase* in water intake over the course of the intervention (adjusted pairwise comparison for planned (*P* = 0.001) and performed (*P* = 0.002) engagement).

## Discussion

This exploratory trial was among the first to test the preliminary efficacy of a technology-based mHealth intervention for improving dietary intake among autistic youth who are picky eaters. While this technology intervention produced few between-group differences in the consumption of targeted foods and beverages, subgroup analyses revealed that some children, namely those who consumed few FV at baseline but showed high engagement with the technology, significantly increased their FV intake by the end of the intervention. These findings remained significant when adjusting for taste and smell sensitivity, which was identified as a significant predictor of FV intake among the study sample. Exploratory stratified analyses further revealed that among children who were high consumers of savory snacks at baseline, those in the education group showed a significant *decrease* in calories consumed from savory snacks over time while children in the technology group showed a trend for a significant *increase* in calories consumed from savory snacks. Also, among children in the intervention group who were low consumers of water at baseline, those who showed little engagement with the technology exhibited a significant *increase* in water intake over the course of the intervention.

Contrary to our hypotheses, we did not find a significant group-by-time interaction for any of the primary dietary outcomes. We did, however, find a statistically significant increase in FV intake by 0.4 servings/day (or 19%) in both groups over the course of the intervention. The magnitude of this increase is approximately one third smaller than that seen in behavioral interventions in children with neurotypical development (53, 73, 74) and represents approximately one-sixth of the FV intake recommendations for children of that age (75). The finding that children in the control group also increased their FV intake was unexpected. It is possible that providing families in the control group with a brief nutrition education and printed handout which summarized the dietary targets, having groups matched for research staff contact time, and simply being part of a nutrition research study led to improvements in FV intake among children who did not receive the technology intervention. The finding may also suggest that even low-level efforts and outreach on topics related to public health and nutrition may be useful for making a small but meaningful impact on health behaviors in this population.

With respect to individual differences in children's response to the intervention, we found that children's taste and smell sensitivity significantly predicted their intake of FV for all participants. That is, for each unit of *lower* taste/smell sensitivity, FV intake increased by 0.13 *±* 0.1 servings/day. These data are in agreement with findings from prior research. For example, Coulthard and Blissett (76) reported that children with neurotypical development who were more sensitive to taste and smell stimuli, as measured by the Sensory Profile, consumed fewer FV. A similar relationship between sensory sensitivity and FV intake has been found previously in children who have ASD (77, 78). Thus, promoting healthy eating is likely particularly challenging for children with increased sensory sensitivities. Future interventions should consider intervening at both the level of support for sensory sensitivities (e.g., occupational therapy, exposure and desensitization, sensory-specific diets) and the level of accommodation by parents (e.g., skills training for parents to modify the taste and/or texture of foods and meals through creative preparation and cooking methods).

We further found a significant effect of BMI z-score on intake of SSB indicating that for each unit increase in children's baseline BMI z-score, SSB intake increased by approximately 2 fl oz/day over the course of the intervention. Several systematic reviews and meta-analyses point to a significant positive association between intake of SSB and BMI during childhood (17, 79–81). Even though SSB intake was a dietary target in the current research, the mHealth intervention did not lead to a reduction in SSB. In our stratified analysis, however, we found statistically significant planned and performed engagement-by-time interactions for water intake. Children who drank little water at baseline significantly increased their water intake over the course of the intervention, even in the absence of engaging much with the technology. Given that SSB intake did not decrease over the course of the intervention, it can be assumed that water intake added on but did not replace SSB among children. Additional efforts will be needed for future intervention design and refinement that target the substitution of SSB with water, especially among children with a higher weight status.

In an exploratory analysis, we tested if children in the technology group who were more engaged with the mHealth intervention showed greater improvements in their intake of the target foods and beverages when compared to children who were less engaged with the technology. With respect to healthy target foods and beverages, we found that children who were low consumers of FV at baseline and showed high performed engagement with the technology-based intervention significantly increased their FV intake by 1.5 servings/day over the course of the intervention. Interestingly, we also found that among children who were low consumers of water at baseline but showed little planned or performed engagement with the technology also significantly increased their water intake over the course of the intervention. This suggests that (a) participating in a mHealth nutrition intervention may be particularly useful for children with low baseline intakes of the target foods and (b) having children engage even a little with the technology-based intervention can lead to significant improvements in intake (as was the case with water intake). Indeed, prior research in adults has confirmed that engagement with a mHealth technology was a significant predictor of dietary intake behavior change (82). It will be critically important to identify components of the mHealth intervention, such as increased interactivity, personalization, or individual feedback, which keep children and families engaged with the technology long-term (83).

With respect to unhealthy target foods and beverages, children in the technology group who were (1) high consumers of savory snacks at baseline and (2) showed high planned and performed engagement with the technology exhibited a significant *increase* of 352 calories consumed from savory snacks over the course of the intervention, while children who were not engaged with the technology showed a decrease in calories consumed from savory snacks. Interestingly, we also showed that children in education group, who did not have access to the technology and were high consumers of savory snacks at baseline, showed a significant *decrease* of 131 calories consumed from savory snacks over time. Reducing children's intake of less healthy foods or substituting unhealthy foods for healthy foods may represent a more challenging intervention target. While this intervention showed some improvements in increasing intake of healthy foods and beverages (e.g., FV, water), the benefits of this increased intake will be limited if there is no concomitant decrease in intake of less healthy foods (e.g., SSB, SSS) and may actually increase overall energy intake.

The strengths of the study include the racial/ethnic diversity of our study sample and the inclusion of children with a range in weight status (∼50% with overweight/obesity). To our knowledge, this study is also among the first to test a technology-based nutrition intervention in autistic youth who are picky eaters. The study also had limitations. One, enrollment of study participants fell short of our recruitment goal (83%) and had a higher attrition rate (18%) than anticipated due to the restrictions of the COVID-19 pandemic (e.g., social distancing, stay-at-home orders) which took place towards the later part of the study. Some families were unable to complete their final visits due to stay-at-home orders, and others indicated that priorities had shifted given new childcare, etc. demands. Not reaching our recruitment goal likely impacted the statistical power for the study. While the current analyses should therefore be regarded as exploratory, it still provides important effect size estimates which will help inform future technology-based interventions. Second, the mHealth technology included a larger number of examples for both healthy and less healthy foods and beverages than did the handout for the education group. This may have differentially affected families' food choices. Also, given the fairly short duration and low intensity of the current intervention, it will be important for future studies to determine if any changes seen in children's dietary intake are maintained long-term. In addition, future studies that aim to promote healthier eating and increased water intake should also evaluate gastrointestinal function in children.

In summary, this mHealth nutrition intervention did not yield a significant increase in intake of targeted foods and beverages among children in the technology group relative to a waitlist education group. Subgroup analyses, however, revealed that some children, namely those who consumed few FV at baseline but showed high engagement with the technology, significantly increased their FV intake by the end of the intervention. Future research should test additional strategies to expand the intervention's impact on a wider range of foods while at the same time reaching a broader group of children who have ASD.

## Data Availability

Deidentified data will be made available upon publication to scientists who provide a methodologically sound proposal for use in accomplishing the aims of the approved proposal. Proposals should be sent to the corresponding author at tkral@nursing.upenn.edu.

## Footnotes

^1^A growing literature has highlighted how language and terminology used to describe autism may reflect and further ableist beliefs (1). Particular attention has been paid to whether person-first (e.g., children with ASD) or identity first (e.g., autistic child) language should be used (2, 3). Out of respect for divided opinions among autistic stakeholders themselves, both approaches to terminology are used.
